# Markers of Endothelial Dysfunction Are Attenuated by Resveratrol in Preeclampsia

**DOI:** 10.3390/antiox11112111

**Published:** 2022-10-26

**Authors:** Thaina Omia Bueno-Pereira, Mariana Bertozzi-Matheus, Gabriela Morelli Zampieri, Joelcio Francisco Abbade, Ricardo C. Cavalli, Priscila Rezeck Nunes, Valeria Cristina Sandrim

**Affiliations:** 1 Department of Biophysics and Pharmacology, Institute of Biosciences, Sao Paulo State University (Unesp), Sao Paulo 18618-689, Brazil; 2 Department of Pathology, Medical School, Sao Paulo State University (Unesp), Sao Paulo 18618-689, Brazil; 3 Department of Gynecology and Obstetrics, Faculty of Medicine of Ribeirao Preto, University of Sao Paulo, Ribeirao Preto, Sao Paulo 14049-900, Brazil

**Keywords:** resveratrol, preeclampsia, arginase, nitric oxide, oxidative stress, endothelial cells

## Abstract

Preeclampsia (PE) is characterized by great endothelial dysfunction, decreased nitric oxide (NO) bioavailability, and higher levels of arginase activity. In the present study, we investigated the potential modulatory effects of trans-resveratrol (RSV) on arginase and endothelial dysfunction biomarkers in endothelial cells exposed to plasma from patients with PE and healthy pregnant (HP) women, and umbilical arteries from patients with PE. Human umbilical vein endothelial cells (HUVECs) were incubated with pooled plasma from 10 HP or 10 PE pregnant women and RSV; umbilical arteries from patients with PE were incubated with RSV; intracellular NO and total reactive oxygen species (ROS) levels were assessed using a probe that interacted with these radicals; total arginase activity was evaluated measuring the urea produced; total antioxidant capacity was measured using the ferric reduction ability power (FRAP) assay; and endothelial dysfunction biomarkers were assessed using qPCR in endothelial cells and umbilical arteries. RSV increased NO levels and decreased total arginase activity in endothelial cells incubated with plasma from patients with PE. In addition, RSV increased total antioxidant capacity and downregulated endothelial dysfunction biomarkers, such as intercellular adhesion molecule-1 (*ICAM-1*), von Willebrand factor (*vWF*), and Caspase-3, (*CASP-3*), in endothelial cells and umbilical arteries from PE patients. RSV treatment positively modulated the *_L_*-arginine–NO pathway, decreased arginase activity, and increased antioxidant capacity, in addition to downregulating endothelial dysfunction biomarkers.

## 1. Introduction

Hypertensive disorders of pregnancy constitute one of the leading causes of maternal and perinatal mortality worldwide [1]. Preeclampsia (PE) is a pregnancy disorder associated with recent-onset hypertension (≥140/90 mmHg), which occurs most often after 20 weeks of gestation and often near-term. It is commonly accompanied by proteinuria and target organ dysfunction such as renal failure, impaired liver function, and pulmonary edema [1]. The risk factors associated with the development of the disease include nulliparity, chronic hypertension, gestational diabetes, pre-gestational body mass index (BMI) > 30, maternal age > 35 years, and type 1 and 2 diabetes [2].

Although the cause of PE is not fully understood, the leading hypothesis is abnormal placentation during early pregnancy [3,4]. At some point, placental ischemia causes endothelial dysfunction, leading to an imbalance between endothelium-derived vasodilator and vasoconstrictor factors that maintain vascular homeostasis [5].

Endothelial dysfunction in PE is a major factor that reduces nitric oxide (NO) bioavailability [6]. NO is an important vasoprotective molecule synthesized from *_L_*-arginine in the endothelium, through a family of calcium calmodulin-dependent enzymes called nitric oxide synthase (NOS); most important, in this context, is endothelial nitric oxide synthase (eNOS, EC 1.14.13.39), found mostly in endothelial cells. Reduced NO bioavailability in PE is caused by an oxidative stress environment resulting from the release of vasoactive factors such as arginase (EC 3.5.3.1).

Arginase is a manganese-containing enzyme that catalyzes the final step of the urea cycle to dispose of toxic ammonia by converting *_L_*-arginine to *_L_*-ornithine and urea [7]. Upregulation of this enzyme results in competition with eNOS for L-arginine, which leads to a decrease in the bioavailability of NO [8,9]. Furthermore, our previous study showed that women with PE have different profiles of circulating levels of arginase, correlating with the severity of the disease and its responsiveness to antihypertensive therapy, in addition to the positive correlation of the enzyme with blood pressure levels in PE [10]. In this scenario, a lack of *_L_*-arginine contributes to the uncoupling of eNOS, which consequently produces superoxide instead of NO [11].

Among several enzymes, nicotinamide adenine dinucleotide phosphate (NADPH) oxidase (NOX) and eNOS are the major enzymes producing superoxide in the endothelium [12,13]. Previous studies have shown that NOX contributes to half of the superoxide generated in endothelial cells exposed to plasma from patients with PE [14]. Superoxide may react with NO, generating an excess of peroxynitrite, a molecule found at elevated concentrations in the vasculature of women with PE [15].

Therefore, strategies for attenuating the underlying endothelial dysfunction in PE have been explored. Among the studied drugs is trans-resveratrol (3,4′,5-trihydroxystilbene) (RSV), a stilbenoid present in many plants including grapes, peanuts, and berries [16]. It directly scavenges oxidants, inhibiting NOX activity and preventing eNOS uncoupling in endothelial cells [17,18]. In our previous studies, we have shown that RSV increases NO bioavailability and antioxidant defense by acting on the antioxidant response element (ARE) promoter sequence in endothelial cells exposed to plasma from patients with PE [19].

Therefore, the present study aimed to investigate the effects of treatment of endothelial cells with plasma from patients with PE on factors related to the *_L_*-arginine–NO pathway, such as arginase activity and reactive oxygen species (ROS) formation. In addition, possible RSV-driven modulation of endothelial dysfunction markers was investigated, including intercellular adhesion molecule-1 (*ICAM-1*), von Willebrand factor (*vWF*), and Caspase-3, (*CASP-3*), in endothelial cells and umbilical arteries from patients with PE.

## 2. Materials and Methods

### 2.1. Study Population

The study was approved by the Research Ethics Committee of the Faculty of Medicine of Ribeirão Preto, Brazil (CAAE 37738620.0.0000.5440, approved on 19 October 2020 FMRP–USP) and approved by the Research Ethics Committee of the Botucatu School of Medicine (n° 4,961,945, approved on 9 September 2021) following the principles of the Helsinki Declaration, and all subjects gave written informed consent. Diagnosis criteria of PE were defined by the American College of Obstetricians and Gynecologists, and when, without a history, a pregnant woman developed hypertension (blood pressure ≥ 140/90 mmHg) with or without proteinuria (≥300 mg in 24-h urine) after the 20th week of gestation [1]. All the preeclamptic patients chosen were under antihypertensive treatment. Exclusion criteria were twin pregnancy, hemostatic abnormalities, diabetes mellitus, chronic hypertension, fetal abnormalities, cancer, and cardiovascular diseases. Maternal venous blood samples were collected in tubes containing heparin. The tubes were rapidly centrifuged (3200× *g* for 10 min) at room temperature, and poor platelet plasma samples were stored at −80 °C. For each sample set, 10 plasma samples from healthy pregnant (HP) women and 10 samples from pregnant women with PE were selected.

### 2.2. Cell Culture

Human umbilical vein endothelial cells (HUVEC-EA.hy 926) provided by Rio de Janeiro cell bank (BCRJ, in Brazil) were cultured until they reached 80–90% of confluence and were treated with supplemented culture medium DMEM (Gibco, Waltham, MA, USA) supplemented with 10% (*v*/*v*) fetal bovine serum (FBS), 100 U/mL penicillin, 100 μg/mL streptomycin, and 2 mmol/L *_L_*-Glutamine (Sigma-Aldrich, Saint Louis, MO, USA), at 37 °C and 5% CO_2_. For the following experiments, the endothelial cells were seeded according to the number of cells indicated by each assay, and next, HUVECs were incubated in the presence or absence of a previously standardized concentration of RSV (10 µM/100 µM-Cayman Chemical^®^, Ann Arbor, MI, USA), NOS inhibitor (L-NAME 100 µM Cayman Chemical, Ann Arbor, MI, USA) and NOX inhibitor (Apocynin 100 µM Thermo Fisher Scientific Inc., Waltham, MA, USA) and 10% (*v*/*v*) the pool of plasma for 24 h.

### 2.3. Umbilical Arteries

After delivery, fragments of the umbilical arteries from pregnant women with PE (*n* = 3) of approximately 10 mg were taken, constituting the umbilical artery sample (approximately 1 cm in length). Adipose and connective tissues were surgically removed from the artery and fragmented into two pieces: one was incubated in the presence of 100 µM RSV and the other in the absence; both were in the incubator at 37 °C for 4 h.

### 2.4. Intracellular NO

The method was DAF-FM, a compound essentially non-fluorescent until it reacts with NO, forming fluorescent benzotriazole. The DAF-FM diacetate (4-amino-5-methylamino 2′,7′-difluororescein diacetate) diffuses into cells across the cell membrane. Once inside the cell, it is deacetylated by intracellular esterases, forming highly fluorescent DAF-FM. In short, the culture supernatant (SNC) was removed from all wells, washed with PBS, and incubated for 30 min. After that, the DAF probe was added to each well and read at 485 excitation filters (485/20 filter) and 520 emissions (528/20 filter), with an endpoint sensitivity of 75 in the Synergy 4 equipment (Biotek, Winooski, VT, USA). Each sample test was performed in quintuplicate.

### 2.5. eNOS Uncoupling

To determine eNOS uncoupling as a source of total reactive oxygen species (ROS), cells were exposed to L-NAME (eNOS inhibitor) [20,21]. The production of total ROS by DHE (Dihydroethidium) occurs through the oxidation of this substance by the superoxide anion (O_2_^−^) and its conversion into 2-hydroxyethidium (2-OH^-E+^), a compound that emits a fluorescence proportional to the levels of ROS in the cells. To perform this assay, the supernatant containing the plasma and treatments was discarded, after the addition of PBS. Immediately afterward, 20 μM of the DHE reagent (Sigma-Aldrich, Saint Louis, MO, USA) was added for 30 min at 37 °C in 5% CO_2_. The evaluation of NOX and uncoupled eNOS-dependent ROS production was quantified by fluorescence in a microplate reader with the Synergy 4 equipment (BioTek, Winooski, VT, USA), at 500–530 nm of excitation and 590–620 nm of emission.

### 2.6. Total Antioxidant Capacity

The total antioxidant capacity was performed using the iron reduction assay (Ferric Reducing Antioxidant Power—FRAP), based on the rapid reduction of iron in ferric tripyridyl triazine (Fe(III)-TPTZ) by antioxidants present in the samples, forming ferrous tripyridyl triazine (Fe(II)-TPTZ, a substance with an intense blue color [22]. The working reagent was prepared using 300 mmol/L of acetate buffer, 10 mmol/L o TPTZ/HCl solution, and 20 mmol/L of ferric chloride. In a 96-well plate, 10 μL of the sample was added with 290 μL of the working solution. A ferrous sulfate curve ranging from 0.0312 to 4 mmol was constructed and the plate was incubated for 5 min. The absorbance was then read at 593 nm on the spectrophotometer (Synergy 4, Biotek, Winooski, VT, USA). Data are expressed in μM/L.

### 2.7. Total Arginase Activity

To evaluate arginase activity in endothelial cells, the QuantiChrom^TM^ Arginase Assay Kit (DARG-100) (BioAssay Systems, Hayward, CA, USA) was used. The method uses a chromogen that forms a specifically colored complex with the urea produced in the arginase reaction. The color intensity is directly proportional to the arginase activity in the sample. Briefly, the cells were washed with PBS and removed from the plate, and subsequently, the microtubes were centrifuged at 1000× *g* at 4 °C for 10 min. Cell pellets were lysed for 10 min in 100 μL of 10 mM Tris-HCl (ph 7.4) containing 1 μM pepstatin A, 1 μM leupeptin, and 0.4% (*w*/*v*) Triton X-100. After lysis, the samples were centrifuged at 14,000× *g* at 4 °C for 10 min and the supernatant was used for the assay.

At first, the standard urea solution was prepared by adding 37.5 μL (1 mM) and 37.5 μL dH_2_O into separate wells in the 96-well plate. We then added 30 μL of the sample into two separate wells and prepared the arginase reaction solution by combining four volumes of arginine buffer and one volume of manganese solution. We then added 7.5 μL to one of the sample wells (ODsample), leaving a sample well without the arginine buffer (control sample, ODblank). Subsequently, the plate was incubated at 37 °C for 3 h.

In the second moment, after the 2 h incubation, the urea reagent was prepared by combining equal volumes of reagent A and reagent B. Then, 150 μL of the urea reagent were added to all wells, and 7.5 μL of the arginine buffer were added to the well of the control sample (ODblank). Then, the plate was incubated for 1 h at room temperature. The absorbance reading was performed using a wavelength at 430 nm in a multifunctional plate reader (Synergy 4, BioTek, Winooski, VT, USA).

### 2.8. Reverse Transcriptase Reaction and Real-Time qPCR

To perform real-time PCR (qPCR), we extracted total RNA from HUVECs incubated with plasma and RSV using the TRIzol^®^ reagent (Life Technologies, Carlsbad, CA, USA) following the manufacturer’s recommendations. After, we performed cDNA synthesis from total RNA samples using the High-Capacity cDNA Reverse Transcription Kit (Life Technologies, Carlsbad, CA, USA), following the manufacturer’s instructions. We performed qPCR experiments in triplicate with no cDNA as a negative control, using the QuantStudio 3™ thermocycler (Applied Biosystems, Waltham, MA, USA). We used the Luna SYBR Green Master Mix Universal kit (New England Biolabs, Ipswich, MA, EUA) for the relative expression assays. Each reaction used 8 μL Luna Universal qPCR, 150 nM of each primer (forward and reverse), 2 μL of cDNA (2.5 ng/μL), and a variable amount of nuclease-free water to yield a final volume of 10 μL. The following genes were analyzed: *ICAM-1* (accession number: 3383), *vWF* (accession number: 7450) and *CASP-3* (accession number: 836) and *GAPDH*/*β-actin* (accession number: 2597). KiCqStart™ SYBR^®^ Green Primers of the mentioned genes were purchased from Sigma-Aldrich. The geometric mean from *GAPDH* and the *β-actin* gene was chosen as a reference gene because it was the gene most stable in our samples. The cDNAs were amplified under the following conditions: initial denaturation at 95 °C/1 min, followed by 40 cycles of denaturation at 95 °C/15 s, and annealing at 60 °C/1 min. The analysis of the dissociation curve was performed at the end of each reaction for quality control.

The qPCR analyses were performed by GeneGlobe Data Analysis Center (QIAGEN 2013–2022) and performed in duplicate for each sample.

### 2.9. Statistical Analyses

Replicates of five per group, combined with treatments (plasma, RSV, and inhibitors), were performed in each experiment. To compare the two groups, we performed multiple *t*-tests. Grouped analyses were performed using two-way ANOVA followed by Bonferroni’s Multiple Comparison Test. When comparing three or more groups we used one-way ANOVA followed by the Kruskal–Wallis test and Dunn’s multiple comparisons test. Results are expressed in means ± SEM. Statistical analyses were performed using GraphPad Prism 6.0 (GraphPad Software, San Diego, CA, USA) and for all tests, a *p*-value ≤ 0.05 (two-tailed) was considered significant.

## 3. Results

### 3.1. Clinical Parameters of HP and PE Pregnant Women Plasma of the Study

Clinical parameters of plasma used in the in vitro studies of the pregnant women with PE and a healthy pregnant group are presented in Table 1.

No differences were identified in the maternal age parameter between the groups, or in the gestational age at sampling. The pregnant women also had similar parameters regarding race, primiparity, and smoking. On the other hand, systolic blood pressure (SBP) and diastolic blood pressure (DBP) are increased in the PE group when compared to the HP group (*p* = 0.0040 and 0.0076, respectively), in addition to higher levels of BMI (*p* = 0.0015). Parameters such as hemoglobin, hematocrit, platelets, and creatinine did not differ between the groups or were not available in the HP group. Women with PE also gave birth to low-birth-weight newborns and had an Apgar score 1 < 7 (*p* = 0.0157 and 0.0015, respectively), although no difference was observed between the frequency of newborns in the Apgar score 2 (Table 1).

### 3.2. RSV Increases NO Levels and Decreases Arginase Activity in Endothelial Cells Incubated with Plasma from PE Pregnant Women

**Figure 1:**
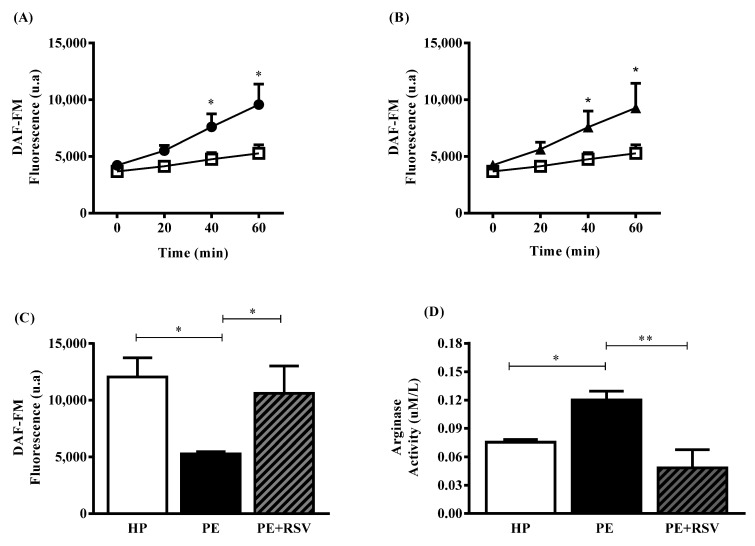
NO fluorescence intensity was measured by DAF-FM for 60 min (**A**–**C**) and arginase activity was quantified according to urea produced (**D**). Human umbilical vein endothelial cells (HUVECs) were incubated with 10% (*v*/*v*) pooled plasma from HP (*n* = 10) and PE (*n* = 10) (**A**) PE group was incubated with RSV (10 µM) for 24 h (**B**) at 60 min of incubation with RSV are shown in (**C**) and arginase activity is demonstrated in (**D**). Data are presented as mean ± SEM. Comparisons between groups by DAF-FM were assessed by two-way ANOVA followed by Bonferroni’s Multiple Comparison Test (A–C) and one-way ANOVA (D) followed by Tukey’s test. * (*p* < 0.05); ** (*p* < 0.001). ● = HP; □ = PE; ▲ = PE + RSV.

Endothelial cells exposed to HP plasma showed higher NO levels when compared to the PE group at 40 and 60 min (*p* < 0.05) (Figure 1A). At the durations, RSV treatment increased NO levels when compared with the PE group in the absence of the antioxidant (*p* < 0.05) (Figure 1B). At 60 min of incubation with the DAF probe, no differences were found between the HP group treated with RSV; however, NO levels presented higher in the HP and RSV groups compared with the PE group (*p* < 0.05) (Figure 1C). Ultimately, endothelial cells exposed to PE plasma presented higher arginase activity levels when compared with the HP group (*p* < 0.05) (Figure 1D). In addition, RSV treatment reversed these high levels when added to the PE group (*p* < 0.001) (Figure 1D).

### 3.3. RSV Combined with eNOS (L-NAME) Inhibitor Decreases DHE Fluorescence, Suggesting Uncoupling eNOS

**Figure 2:**
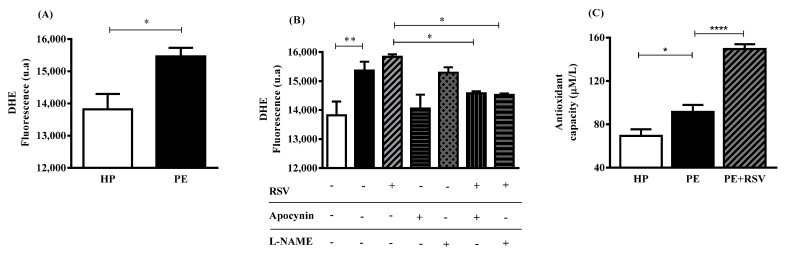
Total ROS fluorescence intensity was measured by DHE (**A**,**B**) and total antioxidant capacity was assessed by FRAP (**C**). Human umbilical vein endothelial cells (HUVECs) were incubated with 10% (*v*/*v*) pooled plasma from HP (*n* = 10) and PE (*n* = 10) (**A**) and then incubated with RSV (10 µM) for 24 h, Apocynin (100 µM) and L-NAME (100 µM) for 30 min before adding the plasma and RSV (**B**) total antioxidant capacity shown in (**C**). Data are presented as mean ± SEM. Comparisons between two groups were performed by the Mann–Whitney test (**A**) Comparisons between three or more groups were performed by one-way ANOVA followed by Tukey’s multiple tests (**B**) and Bonferroni’s multiple tests (**C**). * (*p* < 0.05); ** (*p* < 0.001); ****(*p* < 0.0001).

We found higher levels of DHE fluorescence in the PE group when compared with the HP group (*p* < 0.05) (Figure 2A). RSV treatment did not alter DHE fluorescence in the PE and HP groups (Figure 2B). However, RSV combined with L-NAME and apocynin inhibitors decreased DHE fluorescence intensity when compared with no inhibitors (*p* < 0.05) (Figure 2B).

Regarding antioxidant capacity, endothelial cells incubated with PE plasma showed higher levels of antioxidant capacity when compared with the HP group (*p* < 0.05) (Figure 2C). Interestingly, RSV treatment increased these levels even more (*p* < 0.0001) (Figure 2C).

### 3.4. RSV Downregulates Endothelial Dysfunction Biomarkers in Endothelial Cells Incubated with PE Plasma and Umbilical Arteries from PE Patients

**Figure 3:**
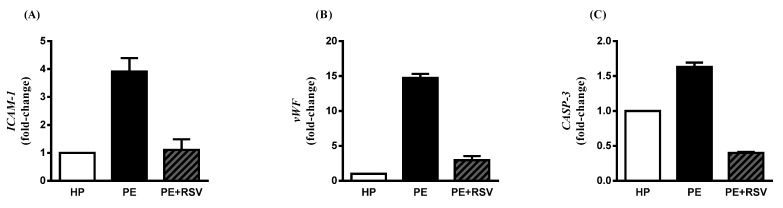
Fold change of *ICAM-1, vWF,* and *CASP-3*. Human umbilical vein endothelial cells (HUVECs) were incubated with 10% (*v*/*v*) pooled plasma from HP (*n* = 10) and PE (*n* = 10) and then incubated with RSV (10 µM) for 4 h (**A**–**C**). Values are presented as fold change ± SEM.

Endothelial cells incubated with plasma from PE women displayed the *ICAM-1* significantly upregulated compared with the HP group (*p* < 0.0001 and fold change > 2.0) (Figure 3A). RSV treatment downregulated the endothelial dysfunction gene significantly, as well as when compared with the PE group (*p* < 0.001 and fold change < 2.0) (Figure 3A).

Regarding *vWF* expression, the PE group showed higher levels when compared with the endothelial cells incubated with HP plasma (*p* > 0.0001 and fold change > 2.0) (Figure 3B). Still, RSV treatment downregulated the endothelial dysfunction gene significantly, as well as when compared with the PE group (*p* > 0.0001 and fold change > 2.0) (Figure 3B).

Finally, *CASP-3* expression in the PE group presents as upregulated when compared to the HP group (*p* < 0.0001 and fold change > 2.0) (Figure 3C). RSV incubation downregulated these levels compared with the PE group without the drug (*p* < 0.0001 and fold change < 2.0).

**Figure 4:**
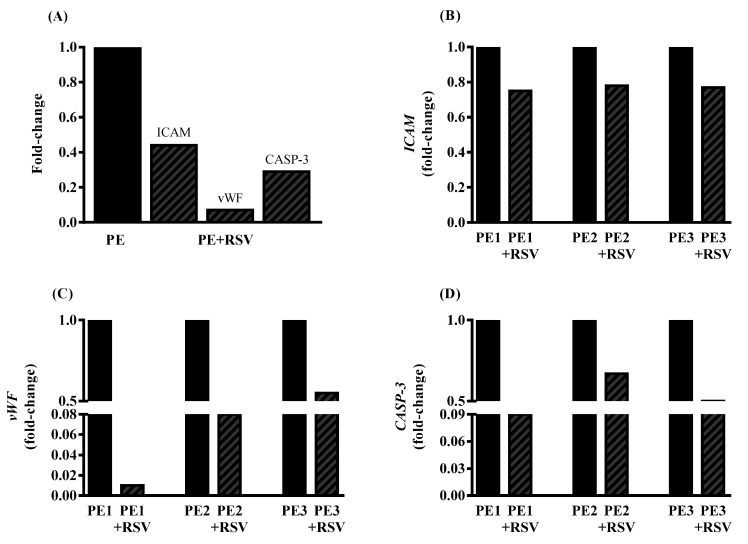
Fold change of *ICAM-1*, *vWF*, and *CASP-3* in all umbilical arteries tested (**A**), followed by independent gene expression in each preeclampsia pregnant individually (**B**–**D**). Umbilical arteries from PE patients were incubated with RSV (100 µM) for 4 h. Values are presented as fold change. Fold change = 1 represents PE without treatment. *p* < 0.05 compared to PE without treatment.

Additionally, we investigated the effect of RSV on umbilical arteries in PE patients. The clinical characteristics of each PE are shown in Table 2. We showed that RSV decreases the expression of *ICAM-1*, *vWF,* and *CASP-3* when compared with umbilical arteries without treatment (fold change < 2) (Figure 4A–D).

## 4. Discussion

Our results demonstrated that RSV treatment significantly modulated the *_L_*-arginine–NO pathway, increasing NO bioavailability and decreasing total arginase activity in endothelial cells incubated with PE plasma. Furthermore, RSV combined with enzyme inhibitors showed even lower levels of total ROS production than RSV combined with PE plasma. In addition, RSV treatment downregulated endothelial dysfunction genes in endothelial cells and umbilical arteries. To our knowledge, this is the first study to show that RSV modulates arginase activity, ROS production, and endothelial dysfunctional genes in an in vitro model of PE.

It is well-defined that healthy pregnant women present higher NO levels than pregnant women with PE [23]. Our data showed that endothelial cells incubated with PE plasma presented decreased NO levels when compared with cells exposed to HP plasma. A reduction in NO bioavailability can be attributed to various agents such as arginase [7]. Arginase plays important roles in vascular diseases and underlying mechanisms related to oxidative stress and inflammation, such as hypertension, atherosclerosis, and ischemic stroke [24,25,26]. In vitro models have provided strong evidence that constitutive that levels of arginase activity in endothelial cells limit NO and NO-dependent vasodilatory function [9,27]. In endothelial cells incubated with PE plasma, increasing arginase levels compete with eNOS for the same substrate (*_L_*-arginine), reducing its levels, which consequently limits the capacity of eNOS to synthesize NO [28]. Taken together, our results corroborate previous findings that HUVECs exposed to PE plasma have higher arginase levels than those exposed to HP plasma. Our group also evaluated circulating levels of arginase and found different profiles of enzyme levels depending on the severity of the disease and responsiveness to antihypertensive treatment [10]. However, under arginase upregulation, eNOS becomes uncoupled, leading to oxidative stress and the release of ROS, such as superoxide [29].

Under endothelial dysfunction and ROS release, uncoupled eNOS and NADPH oxidase (NOX) are partially responsible for the oxidative condition found in the cell environment [30]. In this context, we examined the effect of eNOS and NOX inhibitors (L-NAME and apocynin, respectively) and found no differences between the PE group incubated with L-NAME. We suggest that this is because PE plasma is not sufficient to stimulate eNOS activity (Figure 1A,B). Therefore, the activation of enzyme by the RSV would be necessary to observe this effect. However, apocynin decreased total ROS, suggesting that NOX is involved with ROS production in this model, as previous studies showed [14].

Another repercussion of the endothelial dysfunction in PE is the increase of endothelial activation biomarkers such as intercellular adhesion molecule-1 (*ICAM-1*), von Willebrand factor (*vWF*), and Caspase-3 (*CASP-3*) [31,32,33]. Previous studies have shown higher levels of soluble adhesion molecules in pregnant women with PE than in healthy pregnant women, suggesting endothelial damage in PE [34]. The increase in placental expression of *CASP-3* seems to be related to a decrease in defense against apoptosis [32]. Elevated *vWF* expression in pregnant women with PE, when compared to healthy pregnant women, was related to platelet activation [35]. Our data suggest that endothelial cells exposed to plasma from pregnant women with PE upregulate these biomarkers, and further become dysfunctional.

As eNOS quantification was not the aim of our study, we only used a pharmacological tool to inhibit eNOS activity. Furthermore, the results on eNOS protein levels in preeclampsia are controversial. While some authors found reduced concentrations of eNOS in preeclampsia, others showed an increase or undetectable levels. To our knowledge, only one study has evaluated eNOS levels in this model, and no difference was observed between endothelial cells treated with plasma from preeclamptic pregnant or healthy pregnant women [14,36,37,38]. Therefore, quantification of eNOS protein may not be a good indicator of eNOS pathway activation in this in vitro model.

Therefore, for treatment and/management of this syndrome, research has focused on antioxidant compounds. Trans-resveratrol (RSV) is an antioxidant with important effects on the pathophysiology of PE [39]. Our data showed an increase in NO bioavailability and decrease in arginase activity. These results corroborate previous data demonstrating that RSV enhances NO production through multiple mechanisms, such as eNOS activation/expression [18]. The modulatory action on arginase may be due to the presence of a catechol group in the chemical structure of resveratrol, which could enhance its inhibitory activity [40]. In addition, RSV treatment alone did not alter ROS levels. However, when combined with L-NAME or apocynin, ROS levels were reduced, suggesting that eNOS and NOX are the main producers of ROS, as hypothesized above. Interestingly, even with no changes in ROS levels, our data demonstrated that the cells treated with plasma and RSV showed a higher total antioxidant capacity than the PE plasma without treatment. These results corroborate previous data showing that RSV is a ROS scavenger [41]. Nevertheless, we showed that RSV incubation downregulates endothelial dysfunction genes such as *ICAM-1*, *vWF,* and *CASP-3* in endothelial cells and umbilical arteries of patients with PE. These data corroborate with previous studies showing that RSV acts by reducing these genes [42,43,44].

## 5. Conclusions

Our data suggest that RSV treatment increases NO bioavailability and decreases arginase activity, ROS production combined with enzyme inhibitors, and endothelial dysfunction genes in endothelial cells incubated with plasma from patients with PE and umbilical arteries from PE patients. Further understanding of RSV modulatory actions in the *_L_*-arginine–NO pathway using our model can allow, in the future, the development of a treatment for this disease.

## Figures and Tables

**Table 1:** Clinical parameters of the subjects.

Parameters	Healthy Pregnant	Preeclampsia	*p*
*n*	10	10	
Age (years)	24.0 ± 1	26.0 ± 1	0.2011
Caucasian (%)	100	90	0.3161
Primiparae (%)	70	60	0.9581
Smoker (%)	10	0	0.3161
GAS (weeks)	36.6 ± 0.3	34.6 ± 1.3	0.9893
SBP (mmHg)	110.9 ± 3	136.9 ± 6.9	0.0040
DPB (mmHg)	71.2 ± 3.1	85.8 ± 3.7	0.0076
BMI (kg/m^2^)	24.8 ± 0.5	30.3 ± 1.2	0.0015
Uric acid (mg/dL)	ND	9.1 ± 3.2	-
Hemoglobin (g/dL)	12.8 ± 1.2	11.3 ± 2.3	0.0951
Hematocrit (%)	39.0 ± 4.0	34.5 ± 7.0	0.1227
Platelets (×10^3^/mm^3^)	ND	192.9 ± 41.7	-
Creatinine (mg/dL)	ND	0.7 ± 0.1	-
Newborn weight (g)	3175.0 ± 497.9	2053.0 ± 1074	0.0157
Placental weight (g)	572.2 ± 147.4	472.5 ± 147.1	0.3054
APGAR Score 1 (1 min) <7	0.0	10.0	0.0015
APGAR Score 2 (5 min) <7	0.0	0.0	>0.9999

GAS: gestational age at sampling; SBP: systolic blood pressure DBP: diastolic blood pressure; BMI: body mass index; ND: not determined; 24 h-proteinuria (≥300 mg in 24 h urine in healthy pregnant) and (preeclamptic patients present at 2651 ± 1029 mg in 24 h urine). Values are mean ± standard error and frequency (percentage) for categorical variables. Parametric variables were compared by Student *t*-test and non-parametric by Mann–Whitney test. Categorical variables were compared by contingency table.

**Table 2:** Clinical parameters of the umbilical arteries from each PE patients.

Parameters	#PE1	#PE2	#PE3
Age (years)	29	30	26
GAS (weeks)	39	37	37
SBP (mmHg)	140	140	140
DPB (mmHg)	90	90	90
Uric acid (mg/dL)	6.4	3.5	4.2
Platelets (×10^3^/mm^3^)	248	239	288
Creatinin (mg/dL)	0.9	0.6	0.5
Newborn weight (g)	3690	3545	3050
APGAR Score 1 (1 min) <7	9	8	8
APGAR Score 2 (5 min) <7	10	9	8

GAS: gestational age at sampling; SBP: systolic blood pressure DBP: diastolic blood pressure; Values are presented as an absolute number. *ICAM-1*, *vWF* and *CASP-3* values are present by fold change values followed by a *p*-value.

## Data Availability

The data presented in this study are available in the article.
